# The Interaction between LYVE-1 with Hyaluronan on the Cell Surface May Play a Role in the Diversity of Adhesion to Cancer Cells

**DOI:** 10.1371/journal.pone.0063463

**Published:** 2013-05-22

**Authors:** Yan Du, Hua Liu, Yiqing He, Yiwen Liu, Cuixia Yang, Muqing Zhou, Wenjuan Wang, Lian Cui, Jiajie Hu, Feng Gao

**Affiliations:** 1 Department of Molecular Biology Laboratory, Shanghai Sixth People’s Hospital Affiliated with Shanghai Jiaotong University, Shanghai, China; 2 Department of Clinical Laboratory, Shanghai Sixth People’s Hospital Affiliated with Shanghai Jiaotong University, Shanghai, China; University of Patras, Greece

## Abstract

Hyaluronan (HA), a simple disaccharide unit, can polymerize and is considered a primary component of the extracellular matrix, which has a wide range of biological functions. In recent years, HA was found on the surface of tumor cells. According to previous reports, differing HA content on the cell surface of tumor cells is closely related to lymph node metastases, but the mechanisms mediating this process remained unclear. This research intended to study the surface content of HA on tumor cells and analyze cell adhesive changes caused by the interaction between HA and its lymphatic endothelial receptor (LYVE-1). We screened and observed high HA content on HS-578T breast cells and low HA content on MCF-7 breast cells through particle exclusion, immunofluorescence and flow cytometry experiments. The expression of LYVE-1, the lymph-vessel specific HA receptor, was consistent with our previous report and enhanced the adhesion of HA^high^-HS-578T cells to COS-7^LYVE-1(+)^ through HA in cell static adhesion and dynamic parallel plate flow chamber experiments. MCF-7 breast cells contain little HA on the surface; however, our results showed little adhesion difference between MCF-7 cells and COS-7^LYVE-1(+)^ and COS-7^LYVE-1(−)^ cells. Similar results were observed concerning the adhesion of HS-578T cells or MCF-7 cells to SVEC4-10 cells. Furthermore, we observed for the first time that the cell surface HA content of high transfer tumor cells was rich, and we visualized the cross-linking of HA cable structures, which may activate LYVE-1 on lymphatic endothelial cells, promoting tumor adhesion. In summary, high-low cell surface HA content of tumor cells through the interaction with LYVE-1 leads to adhesion differences.

## Introduction

Invasion and metastasis are the most important biological characteristics of malignant tumors. Tumor cell adhesion plays an important role in tumor invasion and metastasis, including the connection between tumor cells themselves and between tumor cells with other cell types. The transfer of tumor cells involves adhesion and separation (adhesion depolymerization). In the early stage of tumor invasion, individual tumor cells are shed from the primary tumor due to adhesion factor loss, which generates the transfer potential of the cancer cells. During the middle stage of invasion, tumor cells that were transferred into the circulation system adhere to vascular endothelial cells and the extracellular matrix. This process involves many adhesion factors and various other factors that promote or separate adhesion such as cell adhesion molecules (CD44, cadherin). This study primarily discusses problems in adhesion involving tumor cells and lymph endothelial cells.

Hyaluronan (HA) is composed of a linear repeat of disaccharide units consisting of D-glucuronic acid and N-acetylglucosamine and is the primary component of the extracellular matrix. Under physiological conditions, HA is primarily distributed in connective tissue with many other proteins to form a large and complicated network that maintains the space between cells such as the mucosa lamina propria and the outer membrane around blood vessels in skin distribution [1], [2]. Many studies have shown that HA affects tumor angiogenesis, metastasis and invasiveness. In vivo studies found that prior to migration, cells increased their HA concentration at their starting location [3], [4]. In addition, HA was found to increase at the invasion edge of breast cancer cells [5], [6] and in the extracellular environment [7], which reorganizes the matrix of invasive tumor cells. A large number of experimental results have shown that aggressive tumors contain high levels of HA and that increased levels of HA in solid tumors are related to poor tumor differentiation and a reduction in the patient survival rate. Previous studies found that increased HA was produced by the surrounding fibroblasts after stimulation by breast cancer cells [8]. However, invasive tumor cells themselves also could synthesize HA at the cell surface. Many studies focus on the correlation of the amount of HA on the tumor cell surface to its metastasis and have found that the ability of tumor cells to transfer was related to their surface HA content [9], [10]. Itano and colleagues [10] intravenously injected breast cells that produce HA and mutant breast cells that could not produce HA into nude mice. They found that mutant clones displayed significant decreases in metastatic ability compared with the parental cells after intravenous (i.v.) injection into syngeneic mice. Expressing mouse hyaluronan synthase 1 (HAS1) by transfection into HAS^−^ cells defective in hyaluronan synthase activity rescued hyaluronan matrix formation as well as hyaluronan production. Lung metastasis after i.v. injection of HAS1 transfectants was also recovered significantly. Many reports have confirmed that HA content on the tumor cell surface was related to the cell transfer speed [9], [10], [11]. Specifically, high levels of surface HA cause cancer cells to transfer quickly, and low HA levels cause tumor cells to transfer slowly.

Existing literature supports the relationship between HA content on the tumor cell surface and tumor lymphatic metastasis. However, the regulation of tumor adhesion, lymphatic metastasis, and transfer speed by cell surface HA still remains unclear. Studies regarding transfer via the blood pathway showed that tumor cells combined with vascular endothelial cell surface-specific composition promotes tumor cell and vascular endothelial cell adhesion [12], [13]. Furthermore, the tumor transfer rate and adhesion rate were positively related. Studies have also found that CD44, a widely expressed endothelial cell surface HA receptor, affected the adhesion of tumor cells and lymphocytes. Lymphatic metastasis field studies confirmed that high surface levels of HA in mouse melanoma cells promoted quick transfer to lymph nodes, and low surface levels of HA in mouse melanoma cells correlated with slow metastasis to lymph nodes [9]. Lymphatic endothelial cells have a specific hyaluronic acid receptor named lymphatic endothelial cell hyaluronic acid receptor 1 (LYVE-1) [14]. LYVE-1 was hypothesized to have an effect that is similar to CD44 on blood endothelial cells, which is facilitated by interacting with tumor cell surface HA and influences tumor cell adhesion. However, the mechanism by which tumor cell surface HA acts on LYVE-1 to affect its adhesion and transfer in tumor lymphatic metastasis remains unclear.

In this study, the biological role of the interaction between LYVE-1 and tumor cell surface HA in cancer cell adhesion was investigated. HS-578T cells that contain high HA on their surface (HA^high^-HS-578T) and MCF-7 cells that have little HA on the surface (HA^low^-MCF-7) were selected. COS-7 cells transfected with LYVE-1 (COS-7^LYVE-1(+)^) and SVEC4-10 cells (a lymph endothelial cell-like cell line) were used as the models to study the adhesion effect between LYVE-1 and tumor cells expressing high or low levels of HA. HS-578T cells and MCF-7 cells, as the upper cells, independently adhere to the sublayer of COS-7^LYVE-1(+)^ cells and COS-7 cells transfected with control pEGFP-N1 vector (COS-7^LYVE-1(−)^) under static and dynamic states. The results showed that HA, present on the surface of tumor cells, mediated the adhesion of tumor cells to COS-7^LYVE-1(+)^ cells and COS-7^LYVE-1(−)^ cells. HA^high^-HS-578T breast cancer cells had greater adherence to COS-7^LYVE-1(+)^ than COS-7^LYVE-1(−)^ cells via the interaction between HA and lymphatic endothelial cell specific hyaluronic acid receptor LYVE-1. Additionally, MCF-7 breast tumor cells that lack cell surface HA basically cannot adhere through HA and LYVE-1 binding. Similar results were also observed concerning the adhesion of HS-578T cells or MCF-7 cells to SVEC4-10 cells.

## Results

### Visualization of the Pericellular Matrix Formation of 6 Human Breast Cancer Cells

**Figure 1:**
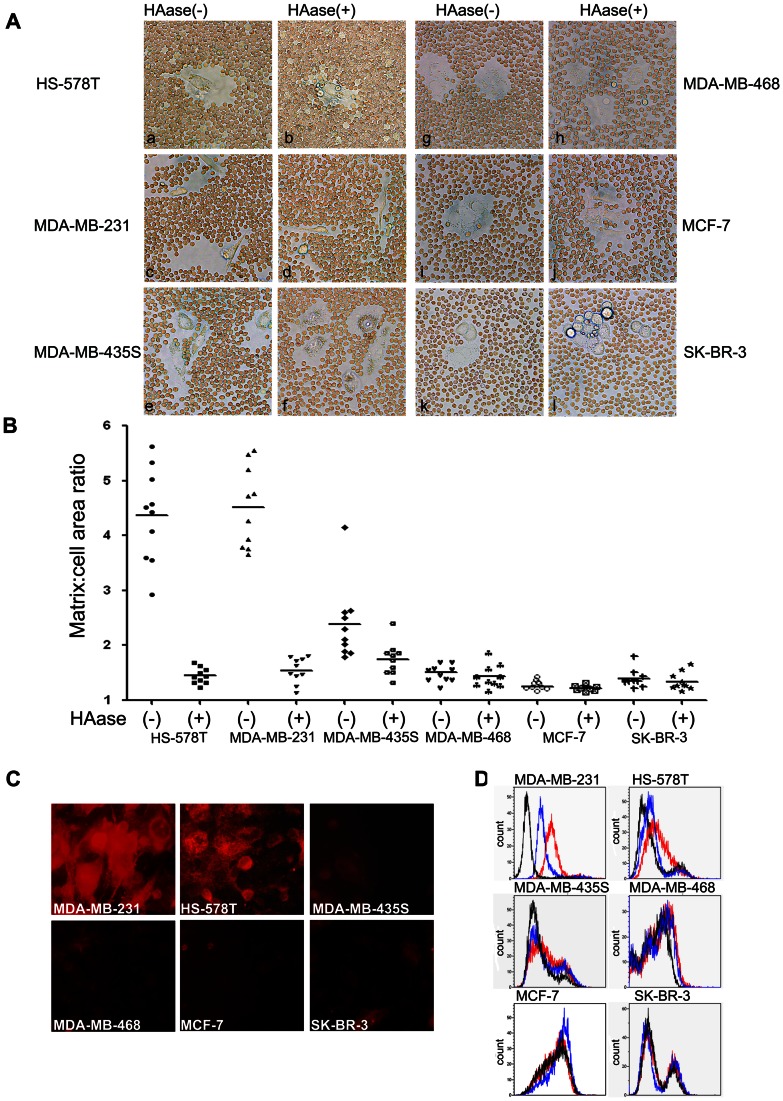
Breast cancer cell surface HA content was detected using three methods. (A) Visualization and morphometric analysis of HA matrices were performed using a particle-exclusion assay before and after the addition of HA-specific Streptomyces hyaluronidase. A clear zone was apparent between HS-578T, MDA-MB-231 cells and red blood cells (a, c); approximately 5 min after Streptomyces hyaluronidase was added to the cells, the HA coats were degraded, allowing the red blood cells to contact the HS-578T and MDA-MB-231 cells (b, d). No difference before (e, g, i, k) and after (f, h, j, l) the addition of Streptomyces hyaluronidase to MDA-MB-435S, MDA-MB-468, MCF-7, and SK-BR-3 cells was observed. (B) Surface HA retention was quantified as the ratio of matrix area to cell area. Individual breast cancer cells were traced using Image Pro Plus 6 at the boundary of the clear zone to calculate the matrix area and at the cell perimeter to calculate the cell area. Coat/cell area ratios are plotted as a distribution, with the mean value represented by a horizontal bar. (C) An immune cell fluorescence experiment was used to detect cell surface HA. The relative brightness represented the HA content on the cell surface. Strong light was visualized in HS-578T and MDA-MB-231 cells; the light of the other four cell types was basically undetectable. (D) HA content was also assessed by FACS analysis. Representative histograms are shown for cells stained with biotinylated HABP and Alexa 488-labeled avidin to HA in untreated cells (red) or cells treated with Streptomyces hyaluronidase (blue). Control cells were stained only with Alexa 488-labeled avidin (black).

The HA content in breast cancer cells differs significantly; therefore, HA present on the surface was visualized using a particle-exclusion assay. In this assay, a clear zone between the HS-578T and MDA-MB-231 cells and the fixed erythrocytes was observed (Fig. 1A a, c). Additionally, 5 min after Streptomyces hyaluronidase was added to the culture dish, HA coats were degraded, allowing the red blood cells to contact HS-578T and MDA-MB-231 cells directly (Fig. 1A b, d). Furthermore, these data are consistent with previous work [15]. Our results showed that MDA-MB-435S, MDA-MB-468, MCF-7, and SK-BR-3 cells, which have a low capacity for HA production, were not excluded from fixed erythrocytes (Fig. 1A e, g, I, k), and almost no change was observed after adding hyaluronidase (Fig. 1A f, h, j, l). To quantify matrix thickness, outlines of matrices and cellular boundaries from 10 individual cells were traced. Coat thickness was plotted for each cell line with and without Streptomyces hyaluronidase treatment (Fig. 1B). The mean ratio for each condition is indicated by a horizontal bar. Consistent with the results of the particle exclusion experiment, immunofluorescence(Fig. 1C)and flow cytometry (Fig. 1D) experiments similarly proved that the HA content on the cell surface of HS–578T and MDA-MB-231 cells was rich, and the other four cells had low surface HA content.

### Breast Cancer Cells HA^high^-HS-578T and HA^low^-MCF-7 Adhere Differently Under Static Conditions

**Figure 2:**
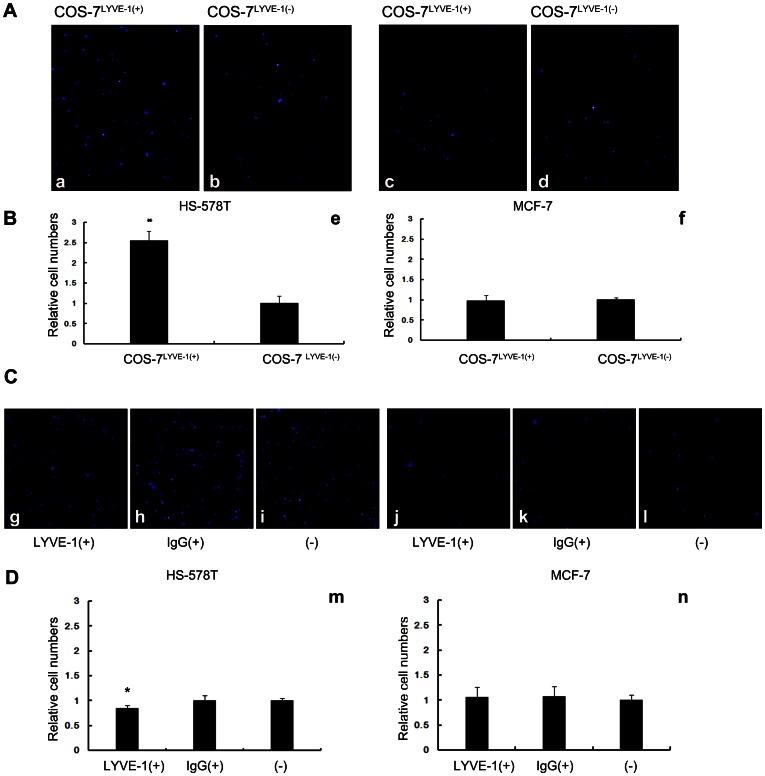
Breast tumor cells expressing high or low levels of HA adhere differently to cells under static conditions. (A) Adhered HS-578T and MCF-7 cells appear as blue spheres (a–d). DAPI-labeled HS-578T cells were more adherent to the confluent monolayer of COS-7^LYVE-1(+)^ cells compared with COS-7^LYVE-1(−)^. MCF-7 cells adhered similarly with both COS-7^LYVE-1(+)^ and COS-7^LYVE-1(−)^ cells. (B) Quantitative evaluations of HS-578T and MCF-7 cell adhesion on the monolayer of COS-7 cells are shown, and each assay was performed in triplicate. The results are shown as the mean ± SD of triplicate wells from a typical experiment and are expressed as a fold increase relative to COS-7 cells transfected with the control vector pEGFP-N1. *P<0.05 versus indicates transfected pEGFP-N1 vector control COS-7 cells. (C) Adhered HS-578T and MCF-7 cells appear as blue spheres (g–l). DAPI-labeled HS-578T cells were adherent to the confluent monolayer of SVEC4-10 cells before and after blocking with LYVE-1 antibody, isotype antibody. (D) Quantitative evaluations of the data from HS-578T and MCF-7 cell adhesion on the monolayer of SVEC4-10 cells are shown, and each assay was performed in triplicate. The results are shown as the mean ± SD of triplicate wells from a typical experiment and are expressed as a fold decrease relative to SVEC4-10 cells without treatment. *P<0.05 versus SVEC4-10 cells without treatment.

As previous studies have shown, the static adhesion experiment is often used to test the adhesion difference between different molecules and cells. To explore the difference in tumor cell adhesion to COS-7 cells through an interaction with surface HA and LYVE-1, a binding assay was performed. The correct expression of LYVE-1 in COS-7 cells was confirmed in our previous study [16]. HA^high^-HS-578T and HA^low^-MCF-7 cells labeled with DAPI were applied to COS-7^LYVE-1(+)^ and COS-7^LYVE-1(−)^ cells to observe stationary adherence. Our results showed that many HS-578T cells adhered to stationary COS-7^LYVE-1(+)^ cells because of the abundance of HA molecules on its surface, and the number of cells that adhered decreased when they interacted with COS-7^LYVE-1(−)^ cells (Fig. 2A, a, b; 2B, e). Previous studies using Streptomyces hyaluronidase before the binding assay showed that HA participates in tumor adhesion [16]. Consistent with this finding, MCF-7 cells, which have low surface HA content, showed no difference in adherence to COS-7^LYVE-1(+)^ cells and COS-7^LYVE-1(−)^ cells (Fig. 2A, c, d; 2B, f). SVEC4-10 cells are verified as a lymph endothelial cell-like cell line [17] and are different from COS-7 cells; therefore, we also tested the adhesion of HS-578T cells and MCF-7 cells to SVEC4-10 cells, which was verified as a lymph endothelial cell-like cell line. Our results showed that after blocking the interaction between LYVE-1 and HA, a slight decrease was observed in the adhesion between HS-578T cells and SVEC4-10 cells (Fig. 2C, g–i; 2D, m). No difference was observed between MCF-7 cells and SVEC4-10 cells (Fig. 2C, j–l; 2D, n). Thus, these findings indicate that the difference noted in the transfer into lymph vessels of tumor cells expressing high and low HA may be due to distinctive adhesion to LYVE-1 on lymph endothelial cells.

### Breast Cancer Cells HS-578T and MCF-7 Differentially Adhere Under Flow Conditions

**Figure 3:**
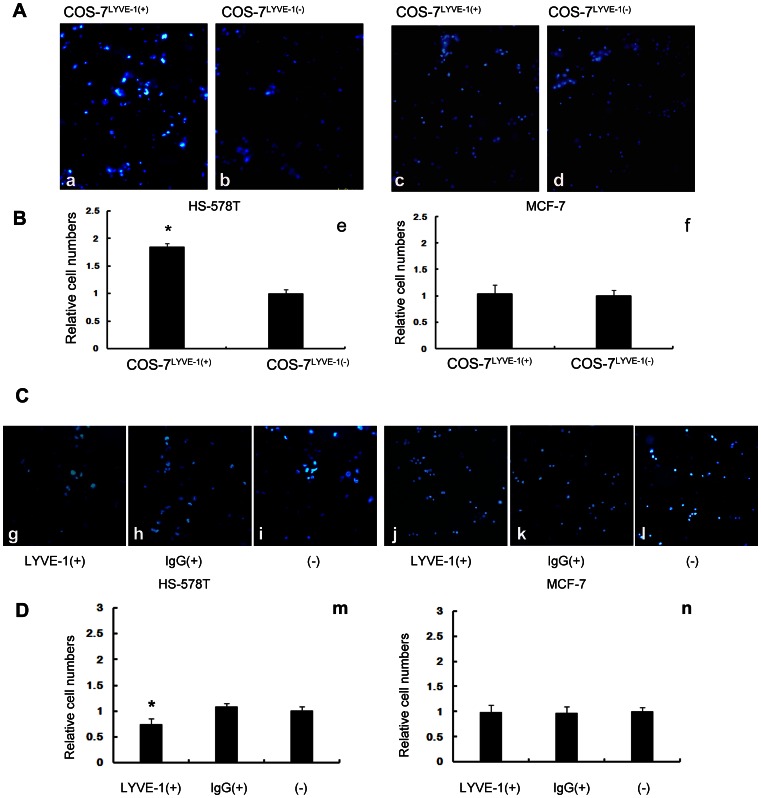
Breast tumor cells expressing high or low levels of HA adhere differently to cells during low flow conditions. (A) The number of HS-578T and MCF-7 cells adhered to COS-7^LYVE-1(+)^ and COS-7^LYVE-1(−)^ cells under 0.1 dyn/cm^2^ low shear stress was evaluated by counting 5 random views of fluorescence microscope images. Adhered HS-578T and MCF-7 cells appear as blue spheres (a–d). (B) Quantitative evaluations of the data representing HS-578T and MCF-7 cell adhesion on the monolayer of COS-7 cells are shown, and each assay was performed in triplicate. The results are shown as the mean ± SD of triplicate wells from a typical experiment and are expressed as a fold increase relative to control pEGFP-N1 vector transfected COS-7 cells. *P<0.05 versus the indicated transfected pEGFP-N1 vector control COS-7 cells. (C) The number of HS-578T and MCF-7 cells adhered to SVEC4-10 cells before and after blocking with LYVE-1 antibody, isotype antibody under 0.1 dyn/cm^2^ low shear stress was evaluated by counting 5 random views of fluorescence microscope images (g–l). (D) Quantitative evaluations of the data from HS-578T and MCF-7 cell adhesion on the monolayer of SVEC4-10 cells are shown. Each assay was performed in triplicate. The results are shown as the mean ± SD of triplicate wells from a typical experiment and are expressed as a fold decrease relative to SVEC4-10 cells without treatment. *P<0.05 versus SVEC4-10 cells without treatment.

Tumor cell arrest and the formation of stable adhesive interactions between tumor cells and endothelial cells are crucial steps in the metastatic process. To gain more information regarding the binding properties of breast cancer cells to COS-7^ LYVE-1 (+)^ and COS-7^LYVE-1 (−)^ cells, similar adhesion experiments to those described above were performed using a parallel plate flow chamber under shear stress conditions. Slides with COS-7^ LYVE-1(+)^ and COS-7^LYVE-1(−)^ cells were placed into a parallel plate flow chamber, and HS-578T or MCF-7 cells were perfused under physiological shear stress. As shown in Fig. 3 (A, a, b; B, e), similar increased adhesion of HS-578T cells to COS-7^LYVE-1(+)^ than COS-7^LYVE-1(−)^ cells was observed under low flow conditions (0.1dyn/cm^2^); however, MCF-7 cells still similarly adhered to both COS-7^LYVE-1(+)^ and COS-7^LYVE-1(−)^ cells (Fig. 3A, c, d; 3B, f). Our results also showed that after blocking the interaction between LYVE-1 and HA, adhesion between HS-578T cells and SVEC4-10 cells slightly decreased under low flow conditions (0.1 dyn/cm^2^), as shown in Fig. 3 (C, g–i; D, m). No obvious differences between MCF-7 cells and SVEC4-10 cells (before and after blocking) were detected (Fig. 3C, j–l; 3D, n).

**Figure 4:**
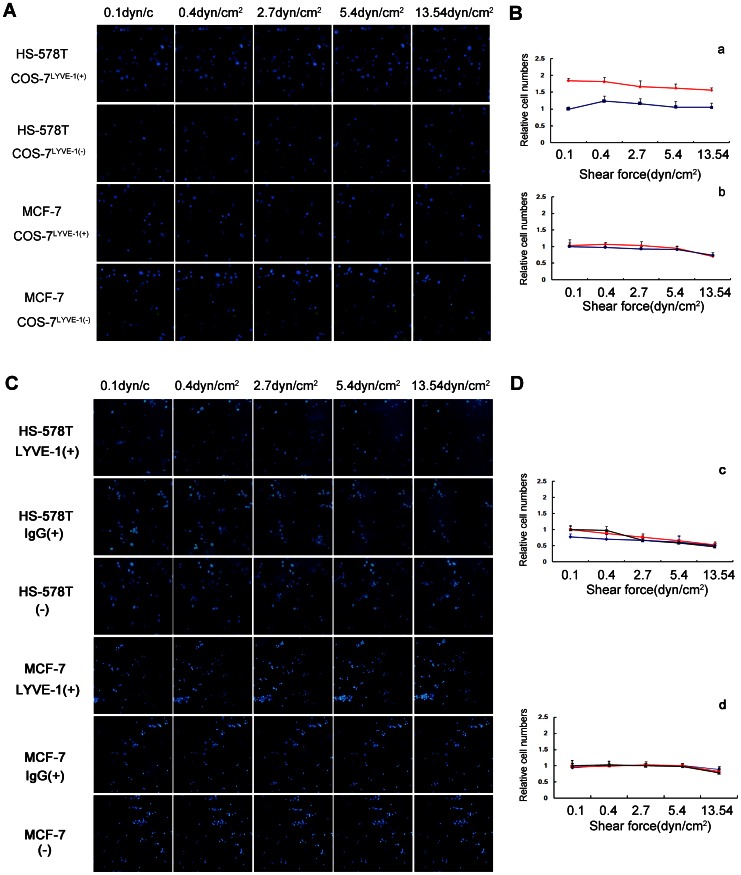
HA-independent adhesion of HS-578T and MCF-7 cells was visualized under flow conditions. HS-578T and MCF-7 cells were perfused over COS-7^LYVE-1(+)^ and COS-7^LYVE-1(−)^ cells at wall shear stresses of 0.1, 0.4, 2.7, 5.4 or 13.54 dyn/cm^2^ for 2 min. (A) The number of adherent cells in the same field is shown as blue spheres. (B) The results are shown as the relative cell numbers of HS-578T cells (a) and MCF-7 cells (b) captured by COS-7^LYVE-1(+)^ (red line) to COS-7^LYVE-1(−)^ (blue line) cells. HS-578T and MCF-7 cells were perfused over a confluent monolayer of SVEC4-10 cells before and after blocking with LYVE-1 antibody, isotype antibody at wall shear stresses of 0.1, 0.4, 2.7, 5.4 or 13.54 dyn/cm^2^ for 2 min. (C) The number of adherent cells in the same field is shown as blue spheres. (D) The results are shown as the relative cell numbers of HS-578T cells (c) and MCF-7 cells (d) captured by SVEC4-10 cells blocking with LYVE-1 (blue line), SVEC4-10 cells blocking with isotype control (red line) to SVEC4-10 cells without treatment (black line). The error bars represent the mean ± SD of the number of cells bound. Data are representative of 3 independent experiments.

The adhesion of HS-578T and MCF-7 cells to COS-7^LYVE-1(+)^ and COS-7^LYVE-1(−)^ cells separately were subjected to different intensities of shear stress, and changes in the number of adhesive cells were measured. HS-578T cells were allowed to bind to LYVE-1-transfected and control cells at 0.1 dyn/cm^2^ for 2 min and were exposed to increasing shear stress of up to 13.54 dyn/cm^2^ (Fig. 4A; B, a). HS-578T cells with high HA content remained bound to the COS-7^LYVE-1(+)^ cell monolayer at wall shear stress levels of up to 5.4 dyn/cm^2^ and began to be released at 13.54 dyn/cm^2^. The amount of HS-578T cell adhesion to COS-7^LYVE-1(+)^ cells compared to COS-7^LYVE-1(−)^ cells was still higher and not obviously changed until the shear stress reached 13.54 dyn/cm^2^. No significant differences in the rolling behavior of MCF-7 cells to COS-7^LYVE-1(+)^ and COS-7^LYVE-1(−)^ cells were observed (Fig. 4A; B, b). However, the adhesion of HS-578T cells to SVEC4-10 cells (before and after blocking) differed from the adhesion to COS-7 cells. A remarkable decrease in adhesion occurred between HS-578T cells and SVEC4-10 cells, which were untreated or treated with IgG_2A_ Isotype control antibody when the shear stress reached 2.7 dyn/cm^2^ (Fig. 4C; D, c). Also, no significant differences in the rolling behavior of MCF-7 cells to SVEC4-10 cells were detected (Fig. 4C; D, d).

### High HA-Expressing Cancer Cells Form Hyaluronan Cable Structures

**Figure 5:**
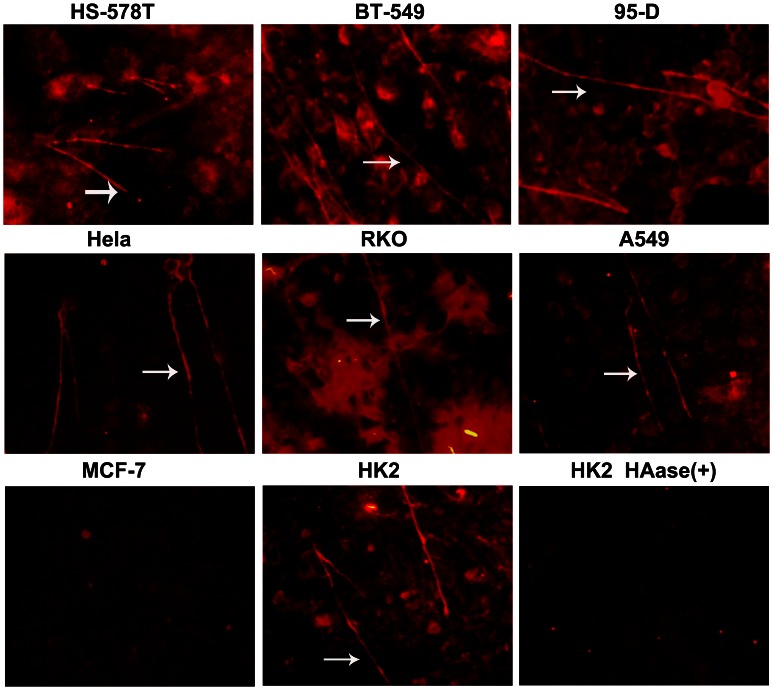
The tumor cell surface HA content is rich, and HA forms cable structures. Confluent monolayer cells were grown in the presence of 10% (v/v) FCS before fixation with methanol and the detection of HA by the addition of bHABP. The sections were imaged by inverted fluorescence microscopy (×20 objective). HA cables are highlighted with white arrows. Original magnification ×20. Streptomyces hyaluronidase was added to the cells before fixation. The HA cables were degraded without detection.

HA cables may have the same capacity to activate LYVE-1 as their capacity to activate CD44 in vivo [18], and our results as well as the findings of previous reports showed that some tumor surfaces contain high levels of HA. However, it was unclear whether HA on the surface of tumor cells develop into cable structures and play a role in activating LYVE-1. Therefore, we attempted to use immunofluorescence to detect HA cables on the surface of tumor cells. Until the tumor cells reached 100% confluence, biotinylated HABP was added into the cells to detect HA distribution. The results showed that the breast cancer cells HS-578T and BT-549, lung cancer 95-D cells, cervical carcinoma HeLa cells, colonic cancer RKO cells, and lung adenocarcinoma A549 cells all contain high levels of HA and develop cable structures (Fig. 5), which were previously reported to occur in HK2 cells. After using Streptomyces hyaluronidase to degrade HA on the tumor surface, the HA cables of HK2 cells were disappeared (Fig. 5). Similarly, the HA cables of the other cells were also abolished by Streptomyces hyaluronidase digestion (data not shown). HA cables were also not detected in MCF-7 cells, which have low levels of surface HA.

## Discussion

This study has shown the HA-mediated mechanism regulating the adhesion of tumor cells to lymph endothelial cells. A significant difference was observed in the adherence of the breast cancer cell line HS-578T, which expresses high levels of HA, with COS-7^LYVE-1(+)^ cells; in contrast, COS-7^LYVE-1(−)^ cells transfected with control vector pEGFP-N1 did not exhibit this alteration under both static and flow conditions. MCF-7 cells lacking HA on their surface adhered in the same manner to COS-7^LYVE-1(+)^ cells as COS-7^LYVE-1(−)^ cells. A slight change was also detected in the adhesion of HS-578T cells to SVEC4-10 cells before and after the addition of blocking antibody. These findings suggest that HA on the tumor surface may participate in breast tumor metastasis by influencing binding with LYVE-1 on lymph vessel endothelial cells.

Early metastasis to lymph nodes is a frequent complication in human breast cancer. According to previous reports, we screened HA on 6 types of breast cancer cells, the highly metastatic cell lines HS-578T [19], [20] and MDA-MB-231 [21], [22], [23], the moderately metastatic cell lines MDA-MB-435S [21], [23] and MDA-MB-468 [24], [25], and the low or no metastatic cell lines MCF-7 [23], [25], [26] and SK-BR-3 [19], [27] through a classical particle exclusion assay, an immunofluorescence experiment, and flow cytometric analysis. Consistent with previous reports [28], we found that HS-578T and MDA-MB-231 cells contain high levels of HA on their surface, while HA levels on the surface of MDA-MB-435S, MDA-MB-468, MCF-7, and SK-BR-3 cells were barely detected. HA on the tumor surface has been shown to be associated with tumor lymph metastasis; therefore, we considered whether and how HA present on the tumor surface participates in this process. To further explore the biological function of HA in tumor lymphatic metastasis, we chose high HA-expressing cells HS-578T and low HA-expressing MCF-7 cells to investigate whether HA inﬂuences the capacity of tumor cells to adhere to COS-7^LYVE-1(+)^ cells and SVEC4-10 cells.

LYVE-1, a cell surface receptor for HA in the lymphatic endothelium, has been proven to be correlated with a high frequency of regional lymph node metastases [29], [30]. Scientists from other laboratories have demonstrated that peritumoral lymph vessels in some types of carcinoma contain tumor emboli decorated with hyaluronan within the lumen, and they suggested that tumor cells within draining lymphatics may bind HA, causing an interaction with the vessel wall via the CD44/HA/LYVE-1 interaction [31]. To directly investigate whether the LYVE-1-HA interaction influences tumor lymph metastasis, we examined the adhesive ability of tumor cells (different HA content) to LYVE-1-positive cells. Our static adhesion assay revealed that LYVE-1 enhances the adhesion of HS-578T cells to COS-7 cells via hyaluronan, which was previously reported [16]. However, whether low HA content in tumor cells produces an interaction with LYVE-1 through other factors to influence tumor cell adhesion is still unclear. We also performed a static adhesion experiment with MCF-7 cells and COS-7^LYVE-1(+)^ and COS-7^LYVE-1(−)^ cells. The present study showed that LYVE-1 did not enhance the adhesion of MCF-7 cells lacking HA on their surface to COS-7 cells. Furthermore, to best model the adhesion of tumor cells to lymph endothelial cells, we used SVEC4-10 cells to repeat the adhesion. Only a slightly decrease was observed after using the blocking antibody, which may be caused by limited interactions with HA and LYVE-1 on SVEC4-10 cells. Taken together, these findings suggest that different levels of HA on tumor cells affect their metastasis into the lymphatic system. The high HA levels on HS-578T cells promote binding to LYVE-1 and adhesion, and the cells may even be pulled into the lymphatic system.

The rolling interaction is a prerequisite for the generation of high strength intermolecular bonds and firm adhesion [32]. To determine the stability of adhesive interactions, hydrodynamic in vitro flow chamber experiments are often utilized [33], [34]. In the present study, to investigate the effect of flow on the adhesiveness of HA^high^-HS-578T cells and HA^low^-MCF-7 cells to COS-7^LYVE-1(+)^ and COS-7^LYVE-1(−)^ cells, the cells were exposed to progressively increasing fluid shear stress. The results showed that HA interactions with LYVE-1 were moderately strong in mediating adhesion under conditions of physiological shear stresses at 2.7 dyn/cm^2^. Firm adhesion was defined as the ability of cells to resist detachment at a wall shear stress of 10 dyn/cm^2^, as previously reported [33]; therefore, the numbers of adhered HS-578T cells decreased as the stress increased to 13.54 dyn/cm^2^, but they still remained partially bound to COS-7^LYVE-1(+)^ cells compared to COS-7^LYVE-1(−)^ cells. Furthermore, the results showed that HA interactions with LYVE-1 on SVEC4-10 cells were weak compared to the interaction with COS-7 cells because the number of adhered HS-578T cells evidently decreased under physiological shear stress at 2.7 dyn/cm^2^. These results suggest that HA promotes a relatively weak and transient adhesion of tumor cells to lymph endothelial cells by interacting with LYVE-1, which may also suggest their roles in tumor cells rolling along the endothelium under lymph flow. As a control, HA^low^-MCF-7 cells were also exposed to COS-7 cells and SVEC4-10 cells, but no significant changes in the adhesion behavior of MCF-7 cells to both cells were observed at any given shear force.

Research conducted by Jackson DG [35] showed that LYVE-1 was functionally “silenced” in a cell-specific manner and that HA-protein complexes such as HA cables may have the same capacity to activate LYVE-1 as the capacity to activate CD44 in vivo [36], [37], [38]. For the first time, the current study provides evidence for the distribution and morphology of HA on tumor cell surfaces. Our study found that HA cables exist on some tumor cell surfaces and that HA is crossed as cables on the cell surface of tumors may activate LYVE-1. However, based on the results published by Jung San Huang, HA stimulates the contraction of the ER network in lymphatic endothelial cells in a LYVE-1-dependent manner [39], and it is believed that HA binds to LYVE-1 in lymph endothelial cells, which was in agreement with our results. These contradictions may be attributed to the different detection methods used in the studies. Nevertheless, HA on the surface of tumor cells may influence tumor adhesion through interactions with the LYVE-1 found on lymphatic endothelial cells.

In conclusion, the results allow us to better understand the mechanism behind the regulation of HA on tumor cell surfaces and the adhesion of tumor cells. HA on tumor cells mediates their adhesion through the lymphatic endothelial cell HA receptor LYVE-1. Adhesion strength depends on the quantity of HA present on the tumor cells; however, this mechanism only plays a role at the beginning of the process, and many questions remain unclear. For example, it is unknown whether tumor cell surface HA is related to the HA synthetic enzyme, the HA degradation enzyme or other molecules. Also, it is unclear whether HA cables activate LYVE-1 on lymph vessels. Further work in this area is ongoing in our laboratory.

## Materials and Methods

### Reagents and Supplies

Biotinylated HABP (hyaluronan binding protein, HABP) was purchased from Merck (Darmstadt, Germany). TRITC-labeled avidin was obtained from Boster (Wuhan, China). Alexa Fluor® 488-conjugated Streptavidin was purchased from Life Technologies (Carlsbad, USA). Mouse LYVE-1 antibody and Rat IgG_2A_ Isotype control were purchased from R&D systems (Minneapolis, USA). DMEM, RPMI-1640, L-15, and F-12k were purchased from Gibco (Stockholm, Sweden). Fetal bovine serum (FBS) was obtained from Hyclone (Lanzhou, China). A Chamlide SC-shear stress chamber was obtained from Live Cell Instruments (Seoul, Korea). All other chemicals were of reagent grade or higher.

### Cell Lines and Culture Conditions

Breast cancer cells (HS-578T, MCF-7), mammalian COS-7 cells (simian virus 40-transformed African Green monkey kidney cell line), and HK-2 cells (human renal proximal tubule cell line) were cultured in Dulbecco’s modified Eagle’s medium. Lung cancer cells (95-D), breast cancer cells (BT-549, SK-BR-3), and SVEC4-10 cells (mouse endothelial cell line, a LEC-like cell line) were cultured in RPMI-1640 medium. Cervical carcinoma cells (HeLa) and colonic cancer cells (RKO) were cultured in Eagle's minimum essential medium. Breast cancer cells (MDA-MB-435S, MDA-MB-231, and MDA-MB-468) were cultured in L-15 medium. Lung adenocarcinoma cells (A549) were cultured in F-12K medium. HK-2 and SVEC4-10 cells were purchased from the American Type Culture Collection. All other cells were purchased from the cell bank at the Shanghai Institute of Cell Biology.

### Plasmid Construction and Transfection

cDNA encoding the lymphatic endothelial cell hyaluronic acid receptor 1 (LYVE-1) was obtained by reverse transcript PCR (RT-PCR) using the plasmid present in our laboratory, and the cDNA was subcloned into the mammalian expression vector pEGFP-N1 as previously described [16]. In brief, for cell surface expression of full-length LYVE-1, the entire LYVE-1 coding sequence was reamplified (94°C for 1 min, 55°C for 1 min, and 72° for 1 min; 30 cycles) from the original clone with LYVE-1 F HindIII (GTACAAGCTTGCCGCCACCATGGCCAGGTGCTTCAGCC) and LYVE-1 R EcoRI (GTCAGAATTCCAACTTCAGCTTCCAGGC) primers using PrimeSTAR HS DNA Polymerase. After digestion with HindIII and EcoRI, the PCR product was ligated into a HindIII/EcoRI-digested pEGFP-N1 vector. The pEGFP-N1 construct was transfected into COS-7 cells using Lipofectamine 2000 (Invitrogen) according to the manufacturer’s instructions.

### Assay for the Presence of HA on the Tumor Cell Surface

**1) Particle-exclusion Experiment.** Breast cancer cells HS-578T, MDA-MB-231, MDA-MB-435S, MDA-MB-468, and MCF-7 as well as SK-BR-3-surface HA matrices were detected using a particle-exclusion assay as previously described [11], [15]. To test HA specificity, 1 U/ml Streptomyces hyaluronidase (HAase) was added after the particles settled. Cell ratios were used to compare coat sizes for different populations. This ratio was defined as the ratio of the area delimited by the perimeter of the coat to the area delimited by the plasma membrane. The relative areas were calculated using Image-Pro Plus 6 software. **2) Immunofluorescence Detection.** The cells were grown on coverslips for 2 days (∼50% confluency) and were first washed with PBS twice and then fixed in 100% methanol at –20°C for 10 min. Next, the coverslips were volatilized until dry, and then the cells were blocked with 1% bovine serum albumin (BSA) in PBS at room temperature for 1 h. The cells were incubated overnight with a 1∶50 dilution of biotinylated HABP 1% BSA-PBS, followed by incubation with a 1∶50 dilution of TRITC-labeled avidin in 1% BSA-PBS for 1 h. The coverslips were then mounted on glass slides. The slides were viewed under an OLYMPUS IX70 inverted phase-contrast fluorescence microscope. Using the same exposure, the number of seconds of exposure allowed for the determination of the relative content of HA. **3) Flow Cytometric Analysis.** The cultured cells were harvested and washed with washing buffer (PBS supplemented with 2% BSA, pH 7.4). A single cell suspension (10^6^/ml) was incubated with or without biotinylated-HABP on ice for 1 h. The cells were washed three times with washing buffer, and 1 µl of the Alexa 488-labeled avidin was added for 1 h on ice. The cells were analyzed in a flow cytometer (Beckman-Coulter, Brea, USA). At least 10,000 cells were analyzed per sample in all experiments, and all experiments were performed at least three times.

### Static Adhesion Assay

The adhesion of HS-578T and MCF-7 cells to LYVE-1-transfected COS-7 cells was measured as previously described [11]. Briefly, COS-7^LYVE-1(+)^ cells and COS-7^LYVE-1(−)^ cells were plated onto 24-well plates in their appropriate medium 3–5 days before the assay and were grown to confluence. On the day of the assay, HS-578T and MCF-7 cells were labeled for 5 min at 37°C with DAPI (Roche, Rotkreuz, Switzerland ) (50 µg/ml) in 1 ml of culture medium. The labeled cells were washed three times with culture medium, counted on a hemacytometer, and resuspended to 10^6^ viable cells/ml adhesion buffers. The labeled cells were added to the monolayer of COS-7 cells. The binding phase of the assay was performed at 37°C for 15 min. Subsequently, the wells were then gently washed three times with adhesion buffer containing DMEM supplemented with 0.1% BSA and fixed in 4% glutaraldehyde in PBS. The number of bound tumor cells was counted under an OLYMPUS IX70 inverted phase-contrast fluorescence microscope.

The adhesion of HS-578T and MCF-7 cells to SVEC4-10 cells was measured as described above. Briefly, SVEC4-10 cells were plated onto 24-well plates before the assay and were grown to confluence. Before the static adhesion assay, the LYVE-1 blocking antibody and its isotype control (10 ug/ml) were added into the wells for approximately 16 h at 37°C. Then, HS-578T and MCF-7 cells were collected and labeled with DAPI and added into the SVEC4-10 cells at 37°C for 15 min. Subsequently, the wells were then gently washed three times with adhesion buffer and fixed in 4% glutaraldehyde in PBS. The number of bound tumor cells was counted using an OLYMPUS IX70 inverted phase-contrast fluorescence microscope.

### Flow Chamber Assay

Parallel plate flow chamber assays were performed as previously described [40]. In brief, COS-7^LYVE-1(+)^ cells, COS-7^LYVE-1(−)^ cells and SVEC4-10 cells (before blocking and after) were isotopically grown to conﬂuent monolayers on glass coverslips in 6-well plates. The glass coverslips were assembled in a parallel Chamlide SC-shear stress chamber and placed on the stage of an inverted phase-contrast microscope. HS-578T and MCF-7 cells were suspended at a density of 10^6^ cells/ml in flow buffer. The chamber was perfused with the cell suspension using a programmable syringe pump (Smiths medical, Hangzhou, China) at room temperature. HS-578T and MCF-7 cells separately accumulated at a shear force of 0.1 dyn/cm^2^ and were exposed to increasing levels of fluid shear (0.1, 0.4, 2.7, 5.4, and 13.54 dyn/cm^2^ each for 2 min). To assess the strength of cell adhesion, the number of adherent cells was determined at the end of each 2-min flow period. At least five fields of microscopic view were analyzed at each shear force.

### Immunofluorescence Detection of HA Cables on the Tumor Cell Surface

The cells were grown on coverslips for 4–5 days (∼100% confluency) and were first washed with PBS twice and then fixed in 100% ice-cold methanol at –20°C for 15 min. Until the coverslips were dry, the cells were blocked with 1% bovine serum albumin (BSA) in PBS at room temperature for 1 h. A 1∶50 dilution of biotinylated HABP in 1% BSA-PBS was then added, and the slides were incubated at 4°C overnight. Next, the slides were washed with PBS before incubation with a 1∶50 dilution of TRITC-labeled avidin for the detection of HA at room temperature for 1 h. Following a final washing step, the coverslips were then mounted onto glass slides, and the slides were viewed under an OLYMPUS IX70 inverted phase-contrast fluorescence microscope. To confirm that HA cables were constructed of HA, Streptomyces hyaluronidase (1 U/ml final concentration) was added for 1 h at room temperature before detection and treatment as described above.

### Statistical Analysis

Statistical analysis was performed using the unpaired Student’s t-test, and a value of P<0.05 was considered to represent a significant difference. The data are presented as the mean±SD. All statistical analysis was performed using SPSS 11.0.
